# Supplementary material for: The impact of news exposure on collective attention in the United States during the 2016 Zika epidemic
Source: PLoS Comput Biol. 2020 Mar 12;16(3):e1007633. doi: 10.1371/journal.pcbi.1007633 (PMC7067377; doi:10.1371/journal.pcbi.1007633)
Supplement: S1 Appendix — SQL code has been used to query the GDELT platform through the Google BigQuery API. (PDF) [file pcbi.1007633.s001.pdf]

```
SELECT
  *
FROM
  [gdelt-bq:gdeltv2.gkg]
WHERE
  DATE > 20160101000000
  AND DATE < 20170101000000
  AND V2Themes LIKE '%TAX_DISEASE_ZIKA%'
  AND V2Locations LIKE '%1#United␣States%'
```
