# Supplementary material for: The impact of news exposure on collective attention in the United States during the 2016 Zika epidemic
Source: PLoS Comput Biol. 2020 Mar 12;16(3):e1007633. doi: 10.1371/journal.pcbi.1007633 (PMC7067377; doi:10.1371/journal.pcbi.1007633)
Supplement: S2 Appendix — (PDF) [file pcbi.1007633.s002.pdf]

Wikipedia pageview scaling

In our modeling framework, we consider a rescaling of page view data by state population. We estimate the scaling dependency of page view data with state population using the approach proposed by Leitão and collaborators [1]. To do so, we use the Python code made publicly available by the authors at the repository: `https://zenodo.org/record/49367#.W23yGP4zbdQ`.

In particular, we estimate the scaling exponent β of the relation

$$PV_s \propto N_s^{\beta} \tag{1}$$

by assuming a Log-normal form of the conditional probability $P(y|x)$, where $y = PV_s$ are total page views by state and $x = N_s$ is the state population.

The best estimate we obtain for the scaling exponent is $\beta = 1.139675 \pm 0.205320$.

References

[1] Leitao JC, Miotto JM, Gerlach M, Altmann EG. Is this scaling nonlinear?. Royal Society open science. 2016 Jul 1;3(7):150649.
