# Supplementary material for: The impact of news exposure on collective attention in the United States during the 2016 Zika epidemic
Source: PLoS Comput Biol. 2020 Mar 12;16(3):e1007633. doi: 10.1371/journal.pcbi.1007633 (PMC7067377; doi:10.1371/journal.pcbi.1007633)
Supplement: S1 Table — Full list of the 128 Wikipedia pages whose page view counts were monitored in the study. The field language refers to the language codes defined by ISO 639-1 and ISO 639-3. (PDF) [file pcbi.1007633.s003.pdf]

	language	page title		language	page title
0	ak	Zika atiridii	34	fa	زیکا ویروس
1	als	Zika-Virus	35	fa	زیکا تب
2	ar	زيكا حمى	36	fi	Zikavirus
3	ar	زيكا فيروس	37	fr	Virus Zika
4	arz	زيكا فيروس	38	frr	Zika-wiirus
5	as	জিকা ভাইৰাছ	39	gl	Virus de Zika
6	ast	Virus del Zika	40	gn	Zika Akãnundu
7	az	Zika virusu	41	he	זיקה □נגיף□
8	bat-smg	Zikas vėrosos	42	hi	जकिा वषिाणु
9	be	Вірус Зіка	43	hr	Zika groznica
10	be	Ліхаманка Зіка	44	ht	Zika
11	bg	Треска Зика	45	hu	Zika-vírus
12	bn	জিকা জ্বর	46	hy	Զիկա ժահր
13	bn	জিকা ভাইরাস	47	ia	Zika virus
14	bs	Zika virus	48	id	Demam Zika
15	ca	Febre de Zika	49	id	Virus Zika
16	ca	Virus del Zika	50	ig	Nje Zika
17	ckb	زیکا ڤایرۆسی	51	it	Virus Zika
18	cs	Zika virus	52	ja	ジカウイルス
19	cy	Y Feirws Zika	53	ja	ジカ熱
20	da	Zikavirus	54	ko	지카열
21	de	Zika-Virus	55	ko	지카바이러스
22	diq	Virusê zika	56	la	Zika virus
23	el	Ασθένεια του ιού Ζίκα	57	li	Zikakoorts
24	el	Ιός Ζίκα	58	lmo	Virus Zika
25	eml	Vìrus Zika	59	lo	ໄວຣັດຊິກາ
26	en	Zika_virus	60	lt	Zika karštinė
27	en	Zika_fever	61	lt	Zika virusas
28	eo	Zika febro	62	lv	Zikas vīruss
29	es	Fiebre del Zika	63	mk	Зика (вирус)
30	es	Virus del Zika	64	ml	സിക്ക വൈറസ്
31	et	Zika-viirusinfektsioon	65	ml	സിക വൈറസ് രോഗം
32	et	Zika viirus	66	mn	Зика вирус
33	eu	Zika birus	67	ms	Demam Zika

	language	page title		language	page title
68	ms	Zika (virus)	102	sr	Зика вирус
69	my	ဇီကာအဖျားရောဂါ	103	sr	Зика грозница
70	my	ဇီကာဗိုင်းရပ်စ်	104	ss	Ifiva ye-Zika
71	mzn	زیکا	105	st	Feberu ya Zika
72	ne	जकिा भाइरस	106	sv	Zikavirus
73	nl	Zikavirus	107	sv	Zikafeber
74	nl	Zikakoorts	108	sw	Homa ya Zika
75	no	Zika-feber	109	ta	ஜிகா வைரஸ்
76	no	Zika-virus	110	te	జికా వైరస్
77	nso	Letadi la Zika	111	th	ไวรัสซิกา
78	ny	Matenda a Zika	112	th	ไข้ซิกา
79	oc	Fèbre de Zika	113	ti	ረስኒ ዚካ
80	oc	Virus de Zika	114	tr	Zika ateşi
81	om	Qandhoon Zikkaa	115	tr	Zika virüsü
82	or	ଜିକା ଭୁତାଣୁ	116	ts	Mukhuhlwana wa Zika
83	or	ଜିକା ଜ୍ୱର	117	uk	Вірус Зіка
84	pa	ਜ਼ੀਕਾ ਵਾਇਰਸ	118	uk	Хвороба, яку спричинює вірус Зіка
85	pl	Wirus Zika	119	ur	زیکا وائرس
86	pt	Vírus da Zika	120	vi	Virus Zika
87	pt	Febre Zika	121	xh	Umkhuhlane i-Zika
88	ro	Febra Zika	122	xmf	ზიკაშ ლახარა
89	ro	Virusul Zika	123	xmf	ზიკაშ ვირუსი
90	ru	Лихорадка Зика	124	yo	Ibà Zika
91	ru	Вирус Зика	125	zh	茲卡熱
92	sco	Zika virus	126	zh	茲卡病毒
93	sh	Zika virus	127	zh-yue	茲卡病毒
94	si	සීකා වෛරසය			
95	simple	Zika fever			
96	simple	Zika virus			
97	sk	Vírus Zika			
98	sl	Mrzlica zika			
99	sl	Virus zika			
100	sn	Zika fivha			
101	sq	Virusi Zika			

Table S1: **Wikipedia pages under study.** Full list of the 128 Wikipedia pages whose page view counts were monitored in the study. The field language refers to the language codes defined by ISO 639-1 and ISO 639-3.
