# Supplementary material for: The impact of news exposure on collective attention in the United States during the 2016 Zika epidemic
Source: PLoS Comput Biol. 2020 Mar 12;16(3):e1007633. doi: 10.1371/journal.pcbi.1007633 (PMC7067377; doi:10.1371/journal.pcbi.1007633)
Supplement: S2 Table — The table reports the Pearson’s correlation coefficient r for the Wikipedia page view counts, the Web news mentioning Zika and the TV close captions at national level. All values of r are statistically significant at p < 10−4. (PDF) [file pcbi.1007633.s004.pdf]

	Wikipedia	News	TV
Wikipedia	1.0	0.74	0.80
News	0.74	1.0	0.78
TV	0.80	0.78	1.0

Table S2: **Correlations between Wikipedia pageviews, the Web news mentioning Zika and TV close captions in 2016.** The table reports the Pearson's correlation coefficient r for the Wikipedia page view counts, the Web news mentioning Zika and the TV close captions at national level. All values of r are statistically significant at $p < 10^{-4}$.
