# Supplementary material for: The impact of news exposure on collective attention in the United States during the 2016 Zika epidemic
Source: PLoS Comput Biol. 2020 Mar 12;16(3):e1007633. doi: 10.1371/journal.pcbi.1007633 (PMC7067377; doi:10.1371/journal.pcbi.1007633)
Supplement: S3 Table — All states are ranked by Pearson’s r values, in descending order. (PDF) [file pcbi.1007633.s005.pdf]

State	Spearman ρ	p-value	Pearson r	p-value	State	Spearman ρ	p-value	Pearson r	p-value
TX	0.76	$< 10^{-3}$	0.748	$< 10^{-3}$	KY	0.59	$< 10^{-3}$	0.204	0.142
AR	0.63	$< 10^{-3}$	0.732	$< 10^{-3}$	NJ	0.59	$< 10^{-3}$	0.192	0.167
VA	0.60	$< 10^{-3}$	0.714	$< 10^{-3}$	ID	0.62	$< 10^{-3}$	0.189	0.176
NE	0.52	$< 10^{-3}$	0.699	$< 10^{-3}$	VT	0.62	$< 10^{-3}$	0.172	0.219
MN	0.59	$< 10^{-3}$	0.662	$< 10^{-3}$	MS	0.59	$< 10^{-3}$	0.163	0.243
CO	0.59	$< 10^{-3}$	0.630	$< 10^{-3}$	KS	0.38	0.005	0.159	0.255
CA	0.66	$< 10^{-3}$	0.629	$< 10^{-3}$	RI	0.38	0.005	0.143	0.307
IL	0.71	$< 10^{-3}$	0.618	$< 10^{-3}$	OH	0.55	$< 10^{-3}$	0.139	0.321
IA	0.49	$< 10^{-3}$	0.617	$< 10^{-3}$	WV	0.48	$< 10^{-3}$	0.127	0.365
MA	0.49	$< 10^{-3}$	0.590	$< 10^{-3}$	OK	0.57	$< 10^{-3}$	0.126	0.370
OR	0.68	$< 10^{-3}$	0.525	$< 10^{-3}$	MO	0.54	$< 10^{-3}$	0.111	0.427
HI	0.71	$< 10^{-3}$	0.519	$< 10^{-3}$	TN	0.48	$< 10^{-3}$	0.110	0.433
FL	0.76	$< 10^{-3}$	0.484	$< 10^{-3}$	WY	0.41	0.002	0.109	0.439
DC	0.66	$< 10^{-3}$	0.476	$< 10^{-3}$	NV	0.52	$< 10^{-3}$	0.102	0.466
NY	0.66	$< 10^{-3}$	0.423	0.002	ND	0.30	0.030	0.095	0.498
WA	0.59	$< 10^{-3}$	0.389	0.004	LA	0.59	$< 10^{-3}$	0.088	0.531
DE	0.55	$< 10^{-3}$	0.353	0.010	IN	0.64	$< 10^{-3}$	0.087	0.537
AL	0.60	$< 10^{-3}$	0.320	0.020	SD	0.42	0.002	0.085	0.543
MD	0.58	$< 10^{-3}$	0.292	0.034	SC	0.46	0.001	0.076	0.587
GA	0.51	$< 10^{-3}$	0.283	0.040	AZ	0.56	$< 10^{-3}$	0.062	0.660
MI	0.48	$< 10^{-3}$	0.269	0.052	NC	0.33	0.017	0.039	0.780
NH	0.68	$< 10^{-3}$	0.262	0.058	ME	0.46	0.001	0.017	0.902
PA	0.54	$< 10^{-3}$	0.261	0.059	NM	0.50	$< 10^{-3}$	0.016	0.909
CT	0.46	$< 10^{-3}$	0.243	0.080	UT	0.39	0.004	0.008	0.955
MT	0.40	0.003	0.205	0.141	WI	0.35	0.011	0.004	0.978

Table S3: **Correlations between Wikipedia pageviews and news mentioning Zika by state**. All states are ranked by Pearson's r values, in descending order.
