# Supplementary material for: The impact of news exposure on collective attention in the United States during the 2016 Zika epidemic
Source: PLoS Comput Biol. 2020 Mar 12;16(3):e1007633. doi: 10.1371/journal.pcbi.1007633 (PMC7067377; doi:10.1371/journal.pcbi.1007633)
Supplement: S4 Table — All states are ranked by Pearson’s r values, in descending order. (PDF) [file pcbi.1007633.s006.pdf]

State	Spearman ρ	p-value	Pearson r	p-value	State	Spearman ρ	p-value	Pearson r	p-value
**MT**	**0.36**	**0.008**	**0.328**	**0.017**	DE	0.00	0.984	-0.082	0.561
WV	0.24	0.087	0.099	0.480	SC	-0.02	0.882	-0.085	0.546
LA	0.11	0.425	0.091	0.517	NV	-0.04	0.786	-0.086	0.541
OK	0.26	0.057	0.009	0.949	KS	-0.01	0.952	-0.090	0.520
AL	0.09	0.505	-0.002	0.990	NJ	0.04	0.801	-0.093	0.506
ID	0.07	0.642	-0.003	0.981	VT	-0.02	0.891	-0.095	0.499
HI	0.14	0.327	-0.005	0.974	MN	0.08	0.590	-0.098	0.486
CT	0.21	0.136	-0.013	0.929	NH	-0.06	0.662	-0.101	0.470
NE	0.06	0.650	-0.014	0.918	DC	-0.08	0.590	-0.102	0.465
MO	0.13	0.371	-0.025	0.861	WA	0.14	0.307	-0.107	0.445
WY	0.00	0.974	-0.035	0.804	OR	-0.05	0.701	-0.115	0.414
NM	0.05	0.730	-0.035	0.802	IN	0.10	0.485	-0.119	0.395
MS	0.17	0.233	-0.036	0.798	WI	-0.05	0.730	-0.121	0.389
TN	0.18	0.187	-0.036	0.796	OH	-0.01	0.967	-0.125	0.374
MI	0.23	0.100	-0.041	0.769	AZ	-0.04	0.760	-0.125	0.373
KY	0.12	0.373	-0.045	0.751	PA	0.14	0.322	-0.125	0.371
FL	0.20	0.161	-0.046	0.741	RI	-0.09	0.538	-0.128	0.361
IL	0.09	0.523	-0.049	0.730	CA	-0.07	0.613	-0.130	0.354
AR	0.06	0.684	-0.057	0.687	UT	-0.12	0.395	-0.132	0.347
SD	-0.03	0.818	-0.063	0.655	NY	0.13	0.354	-0.132	0.346
NC	0.08	0.578	-0.065	0.644	VA	0.08	0.551	-0.132	0.345
IA	0.07	0.613	-0.074	0.601	MD	0.11	0.433	-0.133	0.341
GA	0.13	0.354	-0.074	0.599	CO	0.05	0.737	-0.137	0.327
ND	-0.08	0.549	-0.078	0.577	MA	0.06	0.645	-0.145	0.301
ME	0.10	0.466	-0.079	0.574	TX	0.07	0.640	-0.152	0.278

Table S4: **Correlations between Wikipedia pageviews and ZIKV incidence by state**. All states are ranked by Pearson's r values, in descending order.
