# Supplementary material for: The impact of news exposure on collective attention in the United States during the 2016 Zika epidemic
Source: PLoS Comput Biol. 2020 Mar 12;16(3):e1007633. doi: 10.1371/journal.pcbi.1007633 (PMC7067377; doi:10.1371/journal.pcbi.1007633)
Supplement: S5 Table — All states are ranked by Pearson’s r values, in descending order. (PDF) [file pcbi.1007633.s007.pdf]

State	Spearman ρ	p-value	Pearson r	p-value	State	Spearman ρ	p-value	Pearson r	p-value
AL	0.32	0.020	0.538	$< 10^{-3}$	RI	0.22	0.114	0.057	0.686
TN	0.23	0.097	0.514	$< 10^{-3}$	KY	0.29	0.035	0.056	0.690
NC	0.17	0.212	0.432	0.001	WA	0.19	0.182	0.046	0.742
NY	0.35	0.011	0.431	0.001	MI	0.02	0.863	0.044	0.756
PA	0.35	0.010	0.407	0.002	TX	0.20	0.150	0.026	0.852
SC	0.15	0.297	0.397	0.003	MN	0.15	0.299	0.023	0.869
WV	0.22	0.107	0.372	0.006	AZ	0.08	0.591	0.014	0.923
ID	-0.04	0.802	0.365	0.007	ND	0.00	1.000	0.009	0.950
GA	0.24	0.085	0.351	0.010	OK	0.10	0.463	0.009	0.950
FL	0.42	0.002	0.334	0.014	CO	0.07	0.628	0.009	0.952
WI	0.24	0.079	0.309	0.024	NJ	0.13	0.341	0.001	0.997
IN	0.27	0.052	0.251	0.069	OR	0.03	0.835	-0.000	0.998
IA	0.32	0.022	0.204	0.143	CA	0.01	0.940	-0.006	0.969
MO	0.21	0.140	0.185	0.185	NE	0.11	0.454	-0.022	0.875
UT	0.23	0.100	0.153	0.273	AR	0.18	0.196	-0.025	0.859
KS	0.12	0.410	0.151	0.281	WY	0.00	0.974	-0.040	0.778
MD	0.16	0.247	0.133	0.342	NV	0.04	0.761	-0.041	0.771
MT	0.11	0.424	0.117	0.404	DE	0.02	0.881	-0.042	0.765
VA	0.25	0.076	0.113	0.421	NM	0.05	0.742	-0.046	0.741
MS	0.21	0.124	0.111	0.430	ME	0.07	0.640	-0.059	0.672
DC	0.18	0.186	0.080	0.571	MA	0.16	0.247	-0.063	0.653
HI	0.08	0.571	0.077	0.585	CT	0.09	0.545	-0.077	0.584
IL	0.19	0.183	0.075	0.592	OH	0.14	0.306	-0.078	0.576
NH	0.03	0.853	0.065	0.643	SD	-0.22	0.122	-0.082	0.557
LA	0.24	0.079	0.064	0.649	VT	-0.12	0.379	-0.096	0.493

Table S5: **Correlations between news mentioning Zika and ZIKV incidence by state**. All states are ranked by Pearson's r values, in descending order.
