# Supplementary material for: The impact of news exposure on collective attention in the United States during the 2016 Zika epidemic
Source: PLoS Comput Biol. 2020 Mar 12;16(3):e1007633. doi: 10.1371/journal.pcbi.1007633 (PMC7067377; doi:10.1371/journal.pcbi.1007633)
Supplement: S6 Table — (PDF) [file pcbi.1007633.s008.pdf]

**Features**	a	b	c	d	e
TV	5.70×10^{-8}	-	-	-	-
Web	-	1.42×10^{-8}	-	-	-
TV, Web	4.15×10^{-8}	4.48×10^{-9}	-	-	-
TV, m(TV)	7.93×10^{-8}	-	-	-1.27×10^{-8}	-
Web, m(Web)	-	2.23×10^{-8}	-4.06×10^{-9}	-	-
TV, Web, m(TV)	6.15×10^{-8}	5.41×10^{-9}	-	5.41×10^{-9}	-
TV, Web, m(Web)	4.61×10^{-8}	1.23×10^{-8}	-4.49×10^{-9}	-	-
TV, Web, m(TV), m(Web)	4.59×10^{-8}	1.24×10^{-8}	-4.51×10^{-9}	1.18×10^{-10}	-
TV, Web, m(TV), m(Web), state_news	4.61×10^{-8}	1.20×10^{-8}	-4.55×10^{-8}	2.19×10^{-10}	1.05×10^{-8}

Table S6: **Parameters of the best fitted models for all selected features.** The table reports the values of the fitted coefficients for the best models and the corresponding features. The full model with all the 5 features under consideration takes the following form: $\hat{PV}_s(w) = a \cdot \text{TV}(w) + b \cdot \text{Web}(w) + c \cdot m(\text{Web}) + d \cdot m(\text{TV}) + e \cdot \text{Web}_{state}(w)$.
