# Supplementary material for: The impact of news exposure on collective attention in the United States during the 2016 Zika epidemic
Source: PLoS Comput Biol. 2020 Mar 12;16(3):e1007633. doi: 10.1371/journal.pcbi.1007633 (PMC7067377; doi:10.1371/journal.pcbi.1007633)
Supplement: S7 Table — (PDF) [file pcbi.1007633.s009.pdf]

Table S7: **Comparison of model performance for 49 states and D.C.** For each feature, the average R^2, Pearson r, Spearman ρ, computed over 50 states are reported. Average values of R^2, r and ρ are computed under K-fold cross-validation ($k = 10$). The standard deviation is reported in parenthesis.

**Features**	R^2	Pearson r	Spearman ρ
ZIKV	-0.378 (0.081)	-0.036 (0.029)	0.1366 (0.079)
TV	0.5083 (0.181)	0.7714 (0.098)	0.7633 (0.048)
Web	0.4352 (0.158)	0.7485 (0.096)	0.7702 (0.048)
TV, Web	0.5241 (0.184)	0.7901 (0.100)	0.7898 (0.050)
TV, m(TV)	0.5923 (0.207)	0.8344 (0.107)	0.7599 (0.046)
Web, m(Web)	0.5298 (0.191)	0.7860 (0.101)	0.7018 (0.044)
TV, Web, m(TV)	0.6152 (0.211)	0.8426 (0.108)	0.7700 (0.046)
TV, Web, m(Web)	0.6385 (0.223)	0.8601 (0.109)	0.7747 (0.048)
TV, Web, m(TV), m(Web)	0.6384 (0.223)	0.8600 (0.109)	0.7749 (0.049)
TV, Web, m(TV), m(Web), state_news	0.5080 (0.308)	0.8294 (0.096)	0.7498 (0.037)
