# Supplementary material for: The impact of news exposure on collective attention in the United States during the 2016 Zika epidemic
Source: PLoS Comput Biol. 2020 Mar 12;16(3):e1007633. doi: 10.1371/journal.pcbi.1007633 (PMC7067377; doi:10.1371/journal.pcbi.1007633)
Supplement: Attachment — Submitted filename: Response_to_reviewers.pdf [file pcbi.1007633.s010.pdf]

RESPONSE TO REVIEWERS

Reviewer #1

We thank the reviewer for his positive comments. In the following, we answer to his remarks.

1.1 Although the paper is generally well written, the piece would benefit from some additional copyediting. For example, there appears to be an errant question mark in brackets on page 11 of the PDF. The title also may be somewhat misleading, as I believe this is a study of behavior in the US relative to the Zika outbreak but the 2016 outbreak wasn't limited to the US nor was it really centered in the US, per se. The transmission that actually occurred in the US was quite limited. The authors might just want to revisit how exactly they are framing the phenomenon being studied. There was US transmission but much of the news coverage driving the search behavior reflected international events, unless I am mistaken.

In our study, we focused our attention on the behavior of Americans during the 2016 Zika epidemic. We agree that the title may be misleading and we modified it as: "The impact of news exposure on collective attention in the United States during the 2016 Zika epidemic" to better reflect our scope.

1.2 The authors also miss an opportunity to connect this work to other highly relevant results that have appeared in other journals. Consider, for example, Southwell et al. in Emerging Infectious Diseases, a piece that connects news coverage, search data, and social media data regarding the Zika outbreak discussed here.

We thank the reviewer for pointing us to this relevant reference. We have modified the text of the paper accordingly, to take into account this and other works dealing with news coverage of the Zika outbreak, information seeking behavior and public attention.

As also suggested by other reviewers, we expanded the Introduction and Discussion to better frame our study within the literature of risk communication

1.3 The other major limitation of the paper is the mismatch between theoretically longitudinal concerns with correlational data. Chances are good that time series analysis will tell us a similar story as the correlational analyses here but nonetheless because cases are ordered in time there is a risk that the analysis presented unfairly capitalizes on lurking autocorrelation in the data.

We followed the reviewer's suggestion, as also indicated by other reviewers, and we conducted a time series analysis based on a Vector Autoregression Models (VARS) followed by a Granger causality test. The analysis was conducted using the R package vars: https://rdrr.io/cran/vars

The results of our analysis are reported in a new section of the manuscript, under Results: "Time series analysis".

We first evaluated the optimal lag for a VAR model that is based on the 3 **daily** time series: pageviews, news and TV. We then used the best VAR model (chosen with a lag equal to 8 days according to the Schwartz-Bayes criterion) to evaluate the Granger causality relationship

between the 3 time series. Results indicate that each of the 3 time series Granger-causes the other 2, with a slightly less significant effect of Web news (p-value = 0.018) with respect to the others (p-value<0.001).

On the other hand, based on a Wald-type test, the hypothesis instantaneous causality between each time series and the other two can not be rejected with p-value<10^-6 in all cases.

We then considered **weekly** time series, including the weekly incidence of Zika. A VAR model of the 4 variables with optimal lag of 2 weeks is selected as the best model according to the BIC. A Granger causality test suggests a causal relationship between pageviews and the other 3 variables, and between Web news and the other 3 variables (p<0.05).

The hypothesis of instantaneous causality between pageviews, news and TV, can not be rejected always with p-values<10^-4.

On the other hand, the analysis does not provide support for either a Granger causality nor an instantaneous causality between Zika incidence and the other 3 sources (p-value=0.67).

Overall, the above results support our equal-time regression modeling approach. Now-casting the number of Wikipedia pageviews can be done at state level using data of national media coverage, while their dynamics does not seem to be significantly affected by the incidence of Zika.

Reviewer #2

2.1 Overall, very limited results are presented for the state and city-level analyses. Given the geo-localized Wikipedia pageview available to the authors, the paper could be strengthened by providing timeseries at the state and city level as well as more detailed analyses that showcase the various behavioral responses by geography and language.

We thank the reviewer for the suggestion and we see the limitation of our analysis. Unfortunately, we can not provide a higher resolution analysis of city-level data for two reasons:

- We only retained aggregate page view counts for all languages, but we did not retain pageview time series for each language separately.
- We did not collect pageview timelines for cities but only the cumulative pageview count over a year in each city.

We made the above choices to minimize the security and privacy concerns associated with the release of the dataset.

Although for some languages and some cities the volume of pageview activity is very high, for some other languages and cities pageview counts can be smaller than 10 over a year.
With such small counts, the information is deemed sensitive by our data partner, the Wikimedia Foundation.

We added a sentence in the discussion to highlight such limitation together with further analyses that could be done with higher resolution data.

2.2 The authors considered daily pageviews on 128 different Zika-related Wikipedia articles in 96 languages. However, it is not clear if the Web news captured all 96 languages. Similarly, the TV captions appear to only include English and Spanish. Therefore, it is not clear if the analyses performed are correct given that the different data streams analyzed may be measuring different populations. In short, the same languages should be captured in all the data streams.

We revised the section Data Sources to clarify the language coverage of our datasets. In particular, Web news are captured in 65 different languages, as reported by the GDELT project, and then translated into English. We corrected this point in the corresponding section.

As previously stated in the manuscript, TV captions in our dataset cover 65 different TV stations and 1,140 different TV shows. Indeed, TV shows are only in English and Spanish but we did not limit our query to those languages. The TV Internet Archive includes broadcasting networks in other languages too, however the result of our query returned only broadcasting networks in two languages.

We believe this reflects the actual language coverage of US TV shows. Indeed, according to Wikipedia, TV networks broadcasting in languages other than English and Spanish are a few in

the US and they don’t have a national reach (https://en.wikipedia.org/wiki/Television_in_the_United_States#Other_languages).

2.3 A discussion on why the authors decided to only use Zika and Zika virus for most of the articles analyzed is warranted. Similarly, a justification on why they decided to focus on 43 states with a population in excess of 1 million is needed.

The “Zika fever” and “Zika virus” Wikipedia pages are the only two pages in the English Wikipedia that provide information on the disease (note that “Zika” and “Zika fever” are the same page) and on the pathogen causing the disease (“Zika virus”).

We considered these two, and their counterparts in different languages, because they were by far the most accessed pages in 2016, since the beginning of the international alert. The “Zika virus” page had almost 8 million of worldwide views in 2016, and “Zika fever” had 800,000. The page describing the 2016 outbreak timeline had 300,000 worldwide views but it was created only in May 2016.

We restrict our analysis to the 43 states with at least 1 million inhabitants to remove states where the epidemic activity, considering also imported cases, was negligible for most of 2016 (like Alaska where there were no cases).

Our results are not sensitive to this choice. Now, In the Supplementary Information file, we report also the results of a regression model for all states, excluding Alaska. The performance of the best model slightly decreases but with no significant changes to the overall picture.

2.4 Are duplicate news articles included or removed from the analyses (GDELT)?

Duplicate news articles are removed from GDELT. In our dataset every news item appears only once. We clarified this point in the methods. We also clarified the content of the GDELT dataset, since the description in the original version was ambiguous.

2.5 What is the scaling exponent for city-level analyses? Does the scaling exponent (beta) change for each state or it remains constant across all the states?

We did not analyze the scaling relationship between population size and city-level pageview data because it was not directly relevant to our modelling analysis.

We only computed the scaling exponent for pageview data at state level, thus there is one exponent that describes the relationship between the total volume of pageviews in a state and state population.

Checking whether the scaling relationship holds at city-level and whether the exponent changes in each state would require data from many more cities than we actually have, and we think it would go beyond the scope of our study.

2.6 The authors should consider including tables for the correlations between Wikipedia and TV shows in the supplementary material.

We added Table S3 in the Supplementary Information, summarizing the correlation values presented in the main text.

2.7 It's hard to read the states in Figure 3, consider increasing the font.

The figures were in low resolution. We uploaded high-resolution figures in the revised version.

Reviewer #3

3.1 I suggest you add Sandman's perspective of risk into your literature and into the interpretation of the results.

We thank the reviewer for the suggestion. Indeed, the seminal work by Peter Sandman in 1993 has been one of the first efforts aimed at introducing an emotional and social component in the risk perception of individuals and hence in the risk communication.

In Sandman's view, individual risk is a combination between the actual hazard and the emotional response (in terms of concern, fear or anger). His theory is often exemplified by the equation: Risk = Hazard + Outrage and it was one of the first attempt at ascribing to the public a role in risk communication, since in his view it's the interplay between the external threats and personal (i.e. social and emotional) aspects that shape the meaning of risk.

To include his perspective in the interpretation of our results, we have added the following paragraph in the Discussion:

As originally defined by Sandman, individual perception of risk is the result of a combination between the actual hazard and the emotional response in terms of concern, fear or anger. Sandman's theory is often exemplified by the equation: Risk = Hazard + Outrage and it was one of the first attempt at ascribing to the public a role in risk communication, since in his view it's the interplay between the external threats and personal aspects that shape the meaning of risk. Individual perception of risk during the 2016 ZIKV outbreak has probably decreased quickly, after it became clear that the infection did not pose an immediate threat to most individuals.

3.2 Information seeking - you seem to shift back and forth between collective attention and information seeking. First, I think some additional literature is needed re health info seeking - Nehama Lewis recently wrote a value on information seeking and scanning (2017) for the International Encyclopedia of Media Effects that could be useful. Second, be consistent with your main DV. Do you measure attention to information seeking?

We would like to thank the reviewer for the possibility of clarifying this aspect.

Accesses to Wikipedia pages represent a signal of information seeking behavior, defined as the deliberate process in which individuals actively aim to acquire new knowledge by searching for information on a specific topic, in the words of Nehama Lewis. In our study, we considered such indicator to be a proxy for measuring the collective attention to the outbreak.

We have now added the above paragraph in the Introduction (line 70).

3.3 Your analysis will also benefit from discussing a recent study that looked at the relationships between Zika news coverage and public knowledge, familiarity and information sharing in the US - using a large national survey. The paper is: Ophir & Jamieson (2018). The Effects of Zika Virus Risk Coverage on Familiarity, Knowledge and Behavior in the U.S. – A

Time Series Analysis Combining Content Analysis and a Nationally Representative Survey. This seems especially relevant to your inquiry.

We thank the reviewer for providing us with a relevant reference.

We have included a new paragraph in the Discussion (lines 296-320) to compare our results to those of Ophir and Jamieson. Their results show that the total amount of coverage increased the general population's familiarity and knowledge, more than coverage of specific issues (e.g. microcephaly, sexual transmission etc) related to Zika, while the amount of coverage of specific topics did not predict changes in knowledge. Since in our work we consider information seeking as a proxy for public attention, these findings are consistent with our results.

Moreover, the work of Ophir and Jamieson highlights the different media coverage received by different themes of the ZIKV epidemic. We did not analyze the tones of the news content and we think it would be an important issue to address in further research.

3.4 on p3 - you need to elaborate more on why ZIKV is a communication challenge - the explanations you bring are for why it was a public health challenge, not necessarily a communication one. Use risk or crisis communication literature here.

We would like to thank the reviewer for this comment. Communication about risks related to Zika during the Zika epidemic has been a challenge for public health institutions. In the words of the CDC: "Communicating Zika virus preventive behaviors, transmission paths, and the underlying science are challenging. There are many scientific unknowns, an uncertainty of the risk, and tragic consequences for pregnant women and their fetuses" (https://www.cdc.gov/zika/zap/pdfs/presentations/zap-how-to-communicate-effectively.pdf).

Even the World Health Organization, in 2017, has released a manual (http://www.euro.who.int/en/health-topics/emergencies/zika-virus/technical-reports-and-guidelines-on-zika-virus/emergency-risk-communications/zika-virus-and-emerging-mosquito-borne-diseases-the-european-emergency-risk-communication-challenge.-a-response-guide-2017) on how to deal with the complex communication challenges of Zika and Mosquito Borne Disease outbreaks in general. According to this manual, the Zika experience offers crucial intelligence on what health authorities might expect if the disease were to emerge in their jurisdiction. The main sources of issues for communication listed in the manual are the following:

Dealing with uncertainty: There will be a time-lapse between reports of the first suspected case of Zika-linked microcephaly and the first confirmed case diagnosis of microcephaly is not always easy. The longer this period, the more speculation will occur in the media and among the public.
Increase in information demand: There could be a massive demand for information from the public, media, other health authorities, partners, regulatory entities, and other groups.
Social media exposure: Social media activity may exponentially increase the pressure and demand for information. Social media rumours and misinformation will likely fill gaps in communication by health authorities.

Rumour management: Incomplete and incorrect information and rumours about Zika will emerge among the population and people may adopt measures based on such information.
Managing evolving information: Public health guidance and recommendations may change as more is learned about the outbreak and scientific knowledge about Zika virus and its complications evolve.

We added a paragraph in the Introduction (line 41) to elaborate why ZIKV was a communication challenge, as follows:

The communication challenges posed by ZIKV are well exemplified in a manual released in 2017 by the WHO European Region on how to deal with the complex communication challenges of ZIKV and mosquito borne disease outbreaks in general.
Among the main communication issues listed in the manual, besides Dealing with uncertainty and Rumor management, there are Increase in information demand and Managing evolving information. The last two are particularly interesting as they refer to the massive demand for information from the public, and from the media as well, and to the necessity of adapting public health communication as more is learned about the outbreak and scientific knowledge about Zika virus and its complications evolve.

3.5 p4 - CDC should be Centers for Disease Control and Prevention (add "and Prevention")

We checked and corrected the name of the CDC throughout the manuscript.

3.6 p5 - I'm very concerned with the use of Pearson correlation for variables that will definitely be influenced by autocorrelation. In such case, where I expect autoregression in both observed and unobserved variables that could be correlated with the dependent, I strongly prefer the use of vector autoregression models (VARS). VARS models could be coupled with Granger causality tests to support a causal direction as well.

We followed the reviewer's suggestion, as also indicated by other reviewers, and we conducted a time series analysis based on a Vector Autoregression Models (VARS) followed by a Granger causality test. The analysis was conducted using the R package vars: https://rdrr.io/cran/vars

The results of our analysis are reported in a new section of the manuscript, under Results: "Time series analysis".

We first evaluated the optimal lag for a VAR model that is based on the 3 **daily** time series: pageviews, news and TV. We then used the best VAR model (chosen with a lag equal to 8 days according to the Schwartz-Bayes criterion and Hannan-Quinn criterion) to evaluate the Granger causality relationship between the 3 time series. Results indicate that each of the 3 time series Granger-causes the other 2, with a slightly less significant effect of Web news (p-value = 0.018) with respect to the others (p-value<0.001).

On the other hand, based on a Wald-type test, the hypothesis instantaneous causality between each time series and the other two can not be rejected with p-value<10^-6 in all cases.

We then considered **weekly** time series, including the weekly incidence of Zika. A VAR model of the 4 variables with optimal lag of 2 weeks is selected as the best model according to the BIC. A Granger causality test suggests a causal relationship between pageviews and the other 3 variables, and between Web news and the other 3 variables (p<0.05).

The hypothesis of instantaneous causality between pageviews, news and TV, can not be rejected always with p-values<10^-4.

On the other hand, the analysis does not provide support for either a Granger causality nor an instantaneous causality between Zika incidence and the other 3 sources (p-value=0.67).

Overall, the above results support our equal-time regression modeling approach. Now-casting the number of Wikipedia pageviews can be done at state level using data of national media coverage, while their dynamics does not seem to be significantly affected by the incidence of Zika.

3.7 also on p5 - it has to do with the literature but I really don't think you should have expected a linear relationship between the number of new cases and public reaction. First of all, while it's true that new cases were added, other prior cases were solved... and in almost all cases - Zika came and went without leaving any harm. So it completely makes sense to me that people lost interest in Zika after a while (with an exception for the Rio Olympics where people probably considered its effects on visitors and athletes-- which was also prevalent in news coverage).

It is true that expecting a linear relationship between new cases and public reaction may not be obvious; however, this is a common assumption in most behavior-epidemic models. As rightfully indicated by the reviewer, media coverage will not necessarily depend on the number of cases as well (as discussed below) and media narratives will impact public reaction.

In our study, we wanted to test this assumption at different spatial scales in the US.

We expanded the Discussion to clarify these aspects in relation to previous studies on crisis communication management (lines 301 and following).

3.8 Similarly for the media - we have strong reasons to expect the media NOT to follow the number of cases. Again, I think some literature is missing here - something on newsworthiness (Galtung & Rouge, 1965 or something similar). For various reasons, Zika was just not interesting for journalists. It gained some attention when it was new and mostly unknown, but other than its effects on golfers etc. it wasn't a big deal for journalists. You can

see more about changes in content in Ophir. (2018). Coverage of epidemics in American newspapers through the lens of the Crisis and Emergency Risk Communication Framework.

We agree with the reviewer. Although from a journalist's perspective, it may be obvious that news did not necessarily follow the number of ZIKV cases, our results highlight the limitations of several behavioral epidemic models that incorporate media effects into disease dynamics, as those surveyed in the review by Collinson and Hefferman (2014) Modelling the effects of media during an influenza epidemic. BMC public health, 14(1)..

We added a paragraph in the Discussion (lines 301 and following) to clarify this point.

Moreover, we now extensively cite the works by Ophir, which were clearly an important but missing reference for our study.

3.9 p6 - how reliable do you believe the geographic data is? is it based on ip addresses? these are often inaccurate

Geo-location of page view data is based on IP addresses and it is done by the Wikimedia Foundation using the MaxMind database (https://wikitech.wikimedia.org/wiki/Analytics/Systems/Cluster/Geolocation).

The MaxMind geolocation service is 99.8% accurate on a country level, 90% accurate on a state level in the US and 86% accurate for cities in the US within a 50 kilometer radius (see: https://support.maxmind.com/geoip-faq/geoip2-and-geoip-legacy-databases/how-accurate-are-your-geoip2-and-geoip-legacy-databases/).

3.10 still on p6 - you measure correlations, but by doing that you assume the public follows the media. While plausible, it could also be that journalists "feel" the public interest and opinion (for example in social media) and are therefore affected by people's attention to the disease. You should be careful about the causal assumption here and explain that it could be the other way around. Again - it would be helpful to use autoregressive models with Granger causality to provide additional support for direction, including the optimal lag.

We agree with the reviewer. From the Granger-causality analysis we performed, as reported in our previous answer, a preferential causal direction in time between the pageview and media sources is not evident.

On the other hand, an instantaneous causal relationship between all variables is strongly supported.

We added a sentence in the Discussion (line 295) about the interpretation of our results.

3.11 p11 - line 254 - there is a question mark where a citation number should appear - "disease similar to ZIKV [?]"

Thank you. We inserted the correct citation: Raude, Jocelyn, et al. Social Science & Medicine 230 (2019): 184-193.

3.12 p12 - I think you're too harsh on yourself saying you didn't measure public behavior. Information seeking is a behavior - you could say that you didn't measure behavior on the individual level, or did not measure health-related behavior, but information seeking is definitely a behavior and Wikipedia seems like a reasonable proxy for that.

We agree with the comment and we revised the sentence accordingly, stating that “We showed Wikipedia data can capture collective attention during outbreaks, however, we did not link such signal with a measure of individual behavioral response or the adoption of health protecting behaviors in the population”.

3.13 I'm worried about your decision to limit articles to those including both Zika and United States - Do you really think all relevant articles will include the term "United States"? For example,- people interested in the Rio Olympics in Brazil will look at articles that do not use the term US. Also - you assume that only articles that explicitly connect the disease to the States will have an impact, which might not be the case. Anyway - I would remove the US condition from your search and look at all US media mentions of Zika. But if you decide to stick with your decision, at least explain why you did so in the discussion section and how it affects your conclusions.

We realized the description of the GDELT dataset was inaccurate and we modified it to reflect the actual definition of the query we used (which was reported in the Supplementary Information file).

More specifically:

- We did not select news only in English, instead Web news are present in all the 65 languages of the GDELT platform and they are automatically translated into English.
- We selected articles that had “ZIKA” as theme (V2Themes) and “United_States” as location (V2Locations). These tags are automatically assigned by the GDELT platform to news items, according to their content. However, each news item can have multiple themes and locations, therefore news items in our dataset can mention multiple countries, not only the United States.
- For the State level analysis, the same article can mention multiple States, therefore the same news item can appear in the timelines of different States.

Unfortunately, GDELT does not allow to select media sources based on the country where they are based. The geographic tagging is limited to the textual content of the items. Removing the US condition from our search would include all media sources worldwide that mentioned Zika. In our opinion, this would result in a too large collection of disparate news items. Given our focus on the United States news coverage, we believe ours to be the best choice.

However, we are aware of the limitations of our approach and we added a sentence in the Discussion to highlight them.

3.14 notice that figures are in low resolution. Please provide sharper versions in the revision (e.g., hard to read states' names, etc).

We uploaded high-resolution figures.
